# Effects of Cell Temperature and Reactant Humidification on Anion Exchange Membrane Fuel Cells

**DOI:** 10.3390/ma12132048

**Published:** 2019-06-26

**Authors:** Van Men Truong, Ngoc Bich Duong, Chih-Liang Wang, Hsiharng Yang

**Affiliations:** 1 Graduate Institute of Precision Engineering, National Chung Hsing University, 145 Xingda Road, South District, Taichung City 402, Taiwan; 2 Innovation and Development Center of Sustainable Agriculture (IDCSA), National Chung Hsing University, 145 Xingda Road, South District, Taichung City 402, Taiwan

**Keywords:** AEMFC, water management, cell temperature, reactant humidification

## Abstract

The performance of an anion exchange membrane fuel cell (AEMFC) under various operating conditions, including cell temperature and humidification of inlet gases, was systematically investigated in this study. The experimental results indicate that the power density of an AEMFC is susceptible to the cell temperature and inlet gas humidification. A high performance AEMFC can be achieved by elevating the cell operating temperature along with the optimization of the gas feed dew points at the anode and cathode. As excess inlet gas humidification at the anode is supplied, the flooding is less severe at a higher cell temperature because the water transport in the gas diffusion substrate by evaporation is more effective upon operation at a higher cell temperature. The cell performance is slightly affected when the humidification at the anode is inadequate, owing to dehydration of the membrane, especially at a higher cell temperature. Furthermore, the cell performance in conditions of under-humidification or over-humidification at the cathode is greatly reduced at the different cell temperatures tested due to the dehydration of the anion exchange membrane and the water shortage or oxygen mass transport limitations, respectively, for the oxygen reduction reaction. In addition, back diffusion could partly support the water demand at the cathode once a water concentration gradient between the anode and cathode is formed. These results, in which sophisticated water management was achieved, can provide useful information regarding the development of high-performance AEMFC systems.

## 1. Introduction

Anion exchange membrane fuel cells (AEMFCs) utilizing an anion exchange membrane (AEM) as a solid polymer ion-conducting membrane have been considered as a viable alternative to proton exchange membrane fuel cells (PEMFCs) due to their potential to adopt a non-precious catalyst and low-cost electrolyte, mitigating the cost concern of fuel cell technology [1,2,3]. The AEMFCs, different from the PEMFCs, also provide the practical advantages of lower overpotential electrode reactions [4,5], lower fuel crossover, a less corrosive environment, and the reduction of carbon monoxide (CO) poisoning [6,7], as well as a wider choice of fuels including hydrogen (H_2_), ammonia (NH_3_), urea, and nitrogen-based fuels [8,9,10] for the requirement of the applications. Compared to PEMFCs, the design and development of AEMFCs are focused on the preparation of a high-performance ionic membrane and non-precious catalysts [11,12,13,14,15,16,17,18,19]. Unfortunately, the performance achieved by AEMFCs has struggled due to the operating conditions, such as the temperature, pressure, and humidification of the reactant gases and the interplay of produced/consumed water in the transport of reactant gases. Particularly, the AEMFC involves the production and consumption of water at the anode and cathode, respectively, which makes it more difficult to control the water management in the AEMFC compared to the PEMFC. 

**Figure 1:**
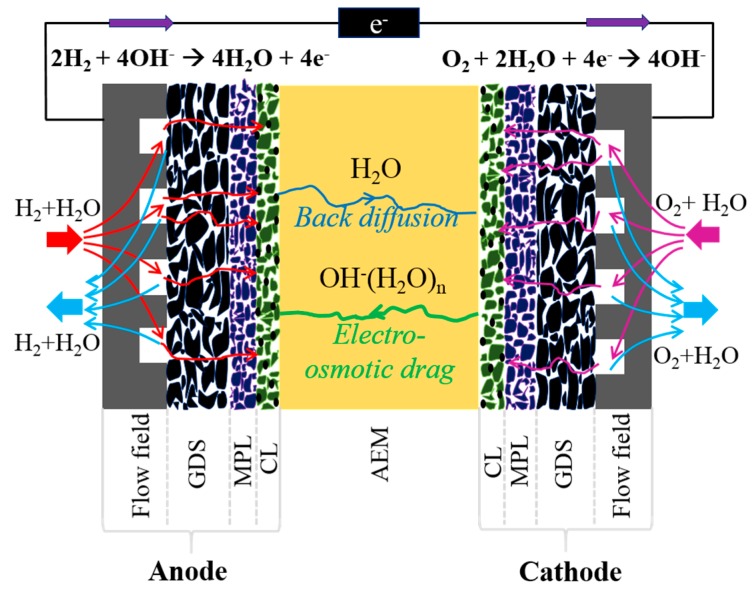
Schematic diagram of a typical anion exchange membrane fuel cell (AEMFC).

A typical schematic diagram of AEMFC, composed of anode/membrane/cathode, involving the conduction medium of OH^−^ within the alkaline membrane is shown in Figure 1. Although the stack design of AEMFC is similar to that of PEMFC, the different conduction medium between the membrane of AEMFC and PEMFC results in their distinct redox. 

Once the electro-osmotic drag moves the water produced at the anode toward the cathode in the PEMFC, the back-diffusion force, built at the water-rich cathode, drives the water back to the water-deficient anode, sustaining membrane hydration and water management during the operating conditions. In contrast, the water balance in AEMFC differs from that in PEMFC. Extra consumption of water in the AEMFC is electrochemically required for the oxygen reduction reaction (ORR) at the cathode. Accordingly, the gradient of intrinsic water content between the two electrodes in the AEMFC is more severe than that of the PEMFC, easily resulting in a dry-out situation at the cathode. On the other hand, the ionic conductivity of the AEM also changes due to the hydration level of the membrane and thereby affects the performance and stability of AEMFCs.

These complicated situations reveal that an in-depth study of water transport inside AEMFCs is urgently required to enable them to be applied in devices. Although the water transport in flow fields can be observed with the visible cell made of transparent materials, it is difficult to visualize the water distribution in the gas diffusion layer (GDL) and catalyst layer (CL) because these layers are formed from opaque materials (e.g., carbon). The neutron-imaging technique has been used to observe the water distribution inside GDL and CL by some researchers, however this technique seems to require further time and spatial resolution [20,21,22]. Thus, an adequate method for capturing water transport in GDL has not been effectively established.

Alternatively, some numerical modeling and experimental works have been proposed to investigate the water transport properties of AEMFCs. For example, Huo et al. [23] developed a three-dimensional multiphase model to investigate water transport in the anode side of AEMFCs. It can be found that the current density and operating temperature have a major influence on the liquid water transport at the anode while the stoichiometric ratio of anode inlet flow has a minor effect on water transport. The removal of the water at the anode can be enhanced through the back-diffusion effect as a thinner membrane is used (around 50 µm). Increasing the porosity of GDL at the anode linearly decreases the amount of liquid water inside the GDL and CL because of a higher porosity which facilitates the removal of water to the flow channel. Similar work has been carried out by Jiao et al. [24]. Their simulation results showed that the mechanism of water removal, depending on the humidification level of the anode, can be a vapor phase (at partial humidification) or liquid phase (at full humidification). Controlled humidification at the cathode is more critical than that at the anode. The liquid water supply at the cathode, particularly during the cell operated at the high current density, has a positive effect on the performance because of the emergent supply of the water from the flow channel and thinner membrane. Deng et al. [25] developed a three-dimensional (3D) half-cell transient model to study the dynamic characteristics of an AEMFC at different steps during operating conditions. Their results indicated that the anode of an AEMFC generally has similar distributions of liquid water, even at different operating stages. The time required for reaching a steady state is related to the current density, operating temperature, and relative humidity of the inlet gases. Dekel et al. [26] presented a new model to investigate the sensitivity of the system over a wide range of material properties, cell designs, and operating parameters in AEMFCs. The effects of the relative humidity (RH) of the gas inlet, operating current density, ionomer loading, and ionomer ion exchange capacity (IEC) on the cell performance can be predicted. Based on an experimental test and analytical modeling, Hou et al. [27] also reported that the performance of an AEMFC can be improved by providing a large pressure gradient between the anode and cathode to alleviate the flooding/drying-out at the anode/cathode and facilitate the reaction kinetics because of the superior permeation of liquid water obtained within the membrane. Oshiba et al. [28] analyzed water transport inside the membrane by changing the thickness of the AEM and the anode flow rate. Their experimental and calculated results revealed that the issue of anode flooding can be suppressed by improving the liquid permeation from the anode to the cathode through using a thin alkaline membrane with a higher flow rate at the anode. Omasta et al. [29] experimentally demonstrated that high-performing AEMFCs can be achieved by optimizing the water balance between the membrane and the electrodes via exploiting operation and design parameters such as the dew point of the gas feed, hydrophobicity of the catalyst layer, gas flow rate of the anode/cathode, flow channel design, and the physicochemical properties of the anion exchange membrane and ionomers. However, this work only focused on the effect of the inlet gas humidification at low cell temperature (60 °C) and may not directly apply for the operation condition at high cell temperature. In addition, although the use of lab-prepared AEM with high ion conductivity (due to a high IEC) and ionomer powder can provide high cell performance and a superior water diffusion rate, which is beneficial for water balance in AEMFCs, the mechanical strength of such a membrane becomes a problem in terms of the membrane durability, limiting the feasibility of the commercialization of AEMFCs.

Although previous studies have made a few efforts regarding water management, systematically experimental studies of the reactant humidification on AEMFCs at different cell temperatures are still few. Tuning the relative humidity (RH) of the feed gases can be a simple and easily controlled way to supply water during cell operation because of the formation of liquid water inside fuel cells. Unfortunately, the electrodes can be flooded at the high RH feed gas while drained of water at the low RH feed gas. These flooding and dry-out situations can increase the mass transport resistance and dehydrate the membrane, respectively, thereby degrading the performance of the AEMFC. On the other hand, the cell temperature also has the tendency to affect the water balance toward flooding/drying-out at the electrodes and the membrane dehydration by altering the behavior of the water transport and gas diffusivity during the cell operation. Accordingly, providing a sophisticated water balance by optimizing the cell temperature and reactant humidification is urgently required to realize a high-performance AEMFC.

In this study, the effects of cell temperature and humidification of the O_2_ and H_2_ reactant gases on the AEMFCs at various cell temperatures were investigated. All of the materials selected for membrane electrode assembly (MEA) were adopted from the commercial products developed for AEMFCs, including Pt/C catalysts, AEM, ionomer, and gas diffusion layers, in order to make our study approach more practical for AEMFC development. The polarization and power density curves were conducted to investigate the water transport of AEMFCs operated at different cell operating temperatures and dew points of the inlet gas stream. The relationship between the optimized dew points and cell temperature is also discussed. Our findings regarding the attainment of an efficient performance can provide useful information for water management in AEMFCs.

## 2. Experimental

In this work, the carbon paper of GDL-30 (supplied by Cetech Co., Ltd., Taichung City 414, Taiwan) was selected as the gas diffusion layers at both the anode and cathode sides, and the preparation was mainly based on the results of our previous study which presented the best cell performance [30]. Basically, the GDL is composed of a gas diffusion substrate (GDS) treated with 30% Polytetrafluoroethylene (PTFE) and a microporous layer (MPL) coated on one side. The GDL properties are also summarized in Table 1. The AEM and ionomer used were AT-1 and aQAPS-S_14_ (supplied by Hephas Energy Co., Ltd., Hsinchu County 30844, Taiwan), respectively, and were primarily developed for AEMFCs. The AT-1 membrane, with a thickness of 30–40 μm, in the dry form, has a specific ion conductivity of ca. 0.1 s cm^−1^ at 60 °C. The similar structure of the AT-1 membrane and aQAPS-S_14_ ionomer facilitated the formation of the ion-aggregating structure, which is beneficial for ion conductivity.

To fabricate gas diffusion electrodes, the catalyst ink was first prepared by mixing the determined amount of Pt/C powder (26.64 mg, 40 wt.% Pt, Tanaka) and 20 wt.% aQAPS-S_14_ ionomer (266.4 mg, 2 wt.% Dimethylformamide, DMF) with a deionized water/IPA (isopropyl alcohol) mixed solution at a volume ratio of 1:1 (300 mL:300 mL) as the dispersant/solvent. Next, the catalyst ink was conducted for 1 h of sonication treatment and was then coated on the MPL surfaces of the GDL-30 by hand-brushing on a hot plate with a temperature of 80 °C. The catalyst was loaded with 0.8 mg cm^−2^ on both the anode and cathode sides. The selection of catalyst and ionomer loadings was based on our preliminary optimization test. The as-received AT-1 membrane was immersed in 1 M KOH solution for more than 48 h at a temperature above 60 °C to replace its original chloride form (Cl^−^) by the hydroxide form (OH^−^). The prepared gas diffusion electrodes were also immersed in 1 M KOH solution for 24 h to change the binder from the Cl^−^ to the OH^−^ form.

To evaluate the cell performance, the prepared AT-1 membrane was sandwiched with two prepared gas diffusion electrodes without hot pressing to form the membrane electrode assembly (MEA). Then, the MEA was installed in a single cell stack composed of two bipolar plates, two gold-coated copper current collectors, and two end plates. Triple serpentine flow fields machined on the bipolar plates had a 1 mm channel width, 1 mm channel height, and 1.5 mm rib width. The active area of the electrodes was 10.24 cm^2^. A couple of 250 µm thick Teflon gaskets (supplied by Hephas Energy Co., Ltd., Hsinchu County 30844, Taiwan ) were placed between the bipolar plates and the MEA to avoid gas leakage and a short circuit. The eight bolts were used to clamp the cell components with 15 kgf cm^−2^ of tightening torque from each bolt.

**Figure 2:**
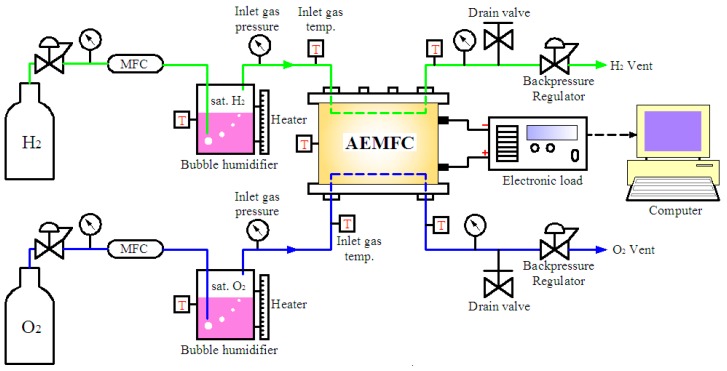
Schematic diagram of the FCED-PD50 test station.

A fuel cell test station (FCED-PD50, Asia Pacific Fuel Cell Technologies, Ltd., Miaoli County 35053, Taiwan) was employed, as illustrated in Figure 2. The inlet gas humidification was tuned by a temperature-controlled bubble humidifier. The gas connections between the gas system and the fuel cell inlets were well-insulated to avoid the condensation of water vapor into liquid water during the delivery. The flow rates of H_2_ and O_2_ were kept constant at 1.0 and 0.5 slm (standard liter per minute), respectively, using the mass flow controller (MFC). All experiments were carried out using the inlet pressure of 30 psi without back pressure. As the single cell stack is ready to test, the cell heating process was gradually implemented by electric heater pads attached on both the anode and cathode sides of the cell from room temperature to 70 °C under flow rates of 0.3 slm for both H_2_ and O_2_ at full humidity. The cell was then activated by operating at 0.75 V under the flow rates of 1.0 slm (H_2_) and 0.5 slm (O_2_) at full humidity until the current density achieved stability. The polarization and power density curves were obtained by applying a varied voltage at a scanning rate of 100 mV/min from an open-circuit voltage (∼1.05 V) to 0.15 V and collecting the corresponding current density.

## 3. Results and Discussion

### 3.1. Effect of Cell Operating Temperature

**Figure 3:**
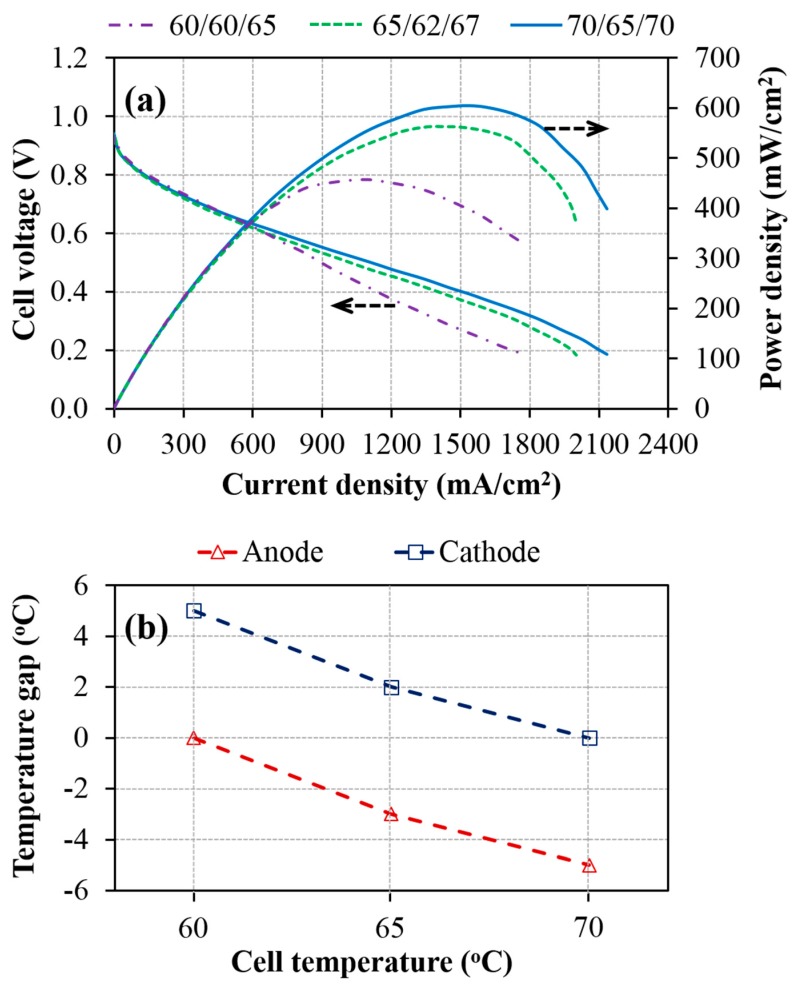
(**a**) Polarization and power density curves of AEMFCs operated at the cell temperatures of 60, 65, and 70 °C with the optimized dew points of the anode/cathode inlet gases at 60 °C/65 °C (60/60/65), 62 °C/67 °C (65/62/67), and 65 °C/70 °C (70/65/70), respectively; (**b**) Temperature gap between optimized dew points and cell temperature (The symbol of 60/60/65 denotes that the cell temperature and dew points of the anode and cathode are 60 °C, 60 °C, and 65 °C, respectively).

Figure 3a shows the performance of the AEMFCs operated at different cell temperatures with the optimized dew points of the anode/cathode inlet gases. The result shows that the optimized cell performance can be gradually improved by increasing the cell operating temperature. Such a trend, which was observed for the cell temperature of 60 °C to 70 °C, can be explained by the temperature dependence of the process parameters in the AEMFC. When the AEMFC is operated at a high cell temperature, the reaction kinetics at the electrodes [31,32] and the membrane ion conductivity [33,34] can be improved. On the other hand, the higher cell operating temperature could also contribute to better water transport in GDS, driven by shear force and evaporation, resulting in less liquid water entrapped inside the GDS and thereby facilitating the permeability of humidified reactant gases and effective water removal during cell operation [35]. However, when the AEMFC is operated at 75 °C, its performance begins to deteriorate after the cycling test due to the failure of the MEA in the AEM degradation.

When comparing the performance of three AEMFCs at different cell temperatures, it can be observed that the temperature gap between the optimized dew point and cell temperature is enlarged at the anode and reduced at the cathode upon raising the cell operating temperature. These temperature gaps at the anode/cathode are 0 °C/5 °C, −3 °C/2 °C, and −5 °C/0 °C, as operated at cell temperatures of 60 °C, 65 °C, and 70 °C, respectively, as shown in Figure 3b. This result indicates that the requirement of inlet gas humidification at the anode and cathode to obtain a desirable performance depends on the cell temperature. This is mainly due to the fact that more water content is carried by higher gas dew points. For example, upon elevating the dew point from 60 °C to 70 °C, the water vapor content in the air will significantly increase from 150–234.8 g m^−3^. In addition, for the AEMFC operated under conditions of high current density, the increase of water production at the anode would partly diffuse to the cathode and take part in ORR. As a consequence, temperature gaps between the optimized dew points at the anode and cathode and the cell temperature are varied with cell temperature. This finding is valuable for those who develop AEMFC with regard to the design of the humidity control system.

### 3.2. Effect of Inlet Gas Humidification

**Figure 4:**
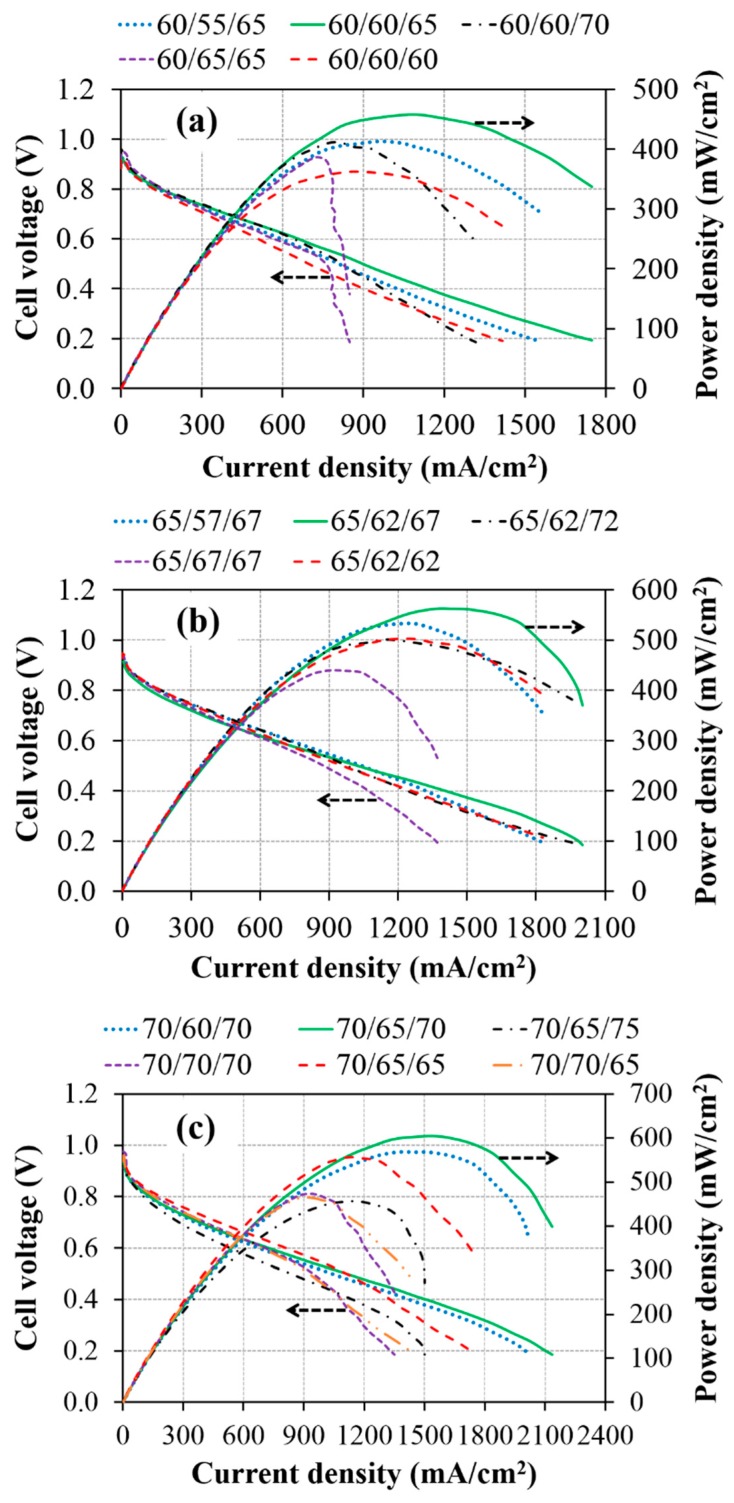
Polarization and power density curves of AEMFCs operated at cell temperature of (**a**) 60 °C, (**b**) 65 °C, and (**c**) 70 °C with different dew points of the anode/cathode inlet gases (the symbol of 60/55/65 denotes that the cell temperature and dew points of the anode and cathode are 60 °C, 55 °C, and 65 °C, respectively).

Figure 4 shows the performance of the AEMFCs operated at different cell temperatures with different dew points of the anode/cathode inlet gases. The experimental results show that the performance of the cells using dew points at either the anode or cathode above/below its optimized values by within 5 °C can be considerably decreased, indicating that the AEMFC performance is highly sensitive to humidification of the feed gases. It is especially found that once the anode humidification at the lower cell temperature is higher than the optimized value, the flooding at the anode side seems to occur easily, resulting in a large reduction in the cell performance (purple dashed curves). This result can be explained by the reduced amount of water removal at a low operating temperature. A higher operating temperature can lead to faster water transport in GDS via evaporation. On the other hand, the performance of AEMFC using low anode humidification could be decreased slightly at a high operating temperature but decreased significantly at a low working temperature (blue dot–dash curves). This behavior can be similarly ascribed to the situation of a lower dew point which leads to lower water vapor content in a gas stream. In addition, the operation of AEMFC at a low operating temperature would generate a lower current density and thereby electrochemically produce a smaller amount of water at the anode, causing the membrane dehydration and thereby the lower membrane ionic conductivity.

Our experimental results also confirm that the supply of water at the cathode inlet is more crucial for the water balance in AEMFC than that at the anode inlet (red curve). This finding is in agreement with reference [27]. The requirement of the water supply in AEMFCs is used to sustain the ORR at cathode electrodes and the hydration of AEMs. In other words, the shortage of water in AEMFCs could directly influence the process of the electrochemical reaction and membrane conductivity, causing a decrease in cell performance. Accordingly, when the humidification at the cathode inlet is lower than the optimized level, the cell performance is considerably reduced, as seen in Figure 4. For example, it is also found in Figure 4c that the operation of AEMFCs using the dew point of 70 °C (70/65/70) and 65 °C (70/65/65) at the cathode has a similar performance at current densities lower than 1000 mA cm^−2^. However, the performance of AEMFC using the dew point of 65 °C decreases substantially at current densities higher than 1000 mA cm^−2^ due to the large demand for water in ORR under high-current conditions. In contrast, the AEMFC that uses the dew point of 75 °C (70/65/75) has an inferior performance compared to that using the dew point of 70 °C (70/65/70). Such a result of the AEMFC using an over humidification supply can be attributed to the mass transport limitation of oxygen at the cathode side because more condensed liquid water is blocking the microporous pores inside the GDL [36,37]. In addition, setting a higher dew point at the cathode will result in a higher water vapor content and a smaller number of O_2_ molecules in the gas stream at the same flow rate, causing fewer O_2_ molecules to be available for ORR and thereby decreasing the cell performance. Similar results can be observed when the cell is operated at lower temperatures (60/60/65)/(60/60/70) in Figure 4a and (65/62/67)/(65/62/72) in Figure 4b.

To further investigate the effect of back-diffusion of water from the anode to the cathode on cell performance, the water concentration gradient between the anode and cathode has been manipulated by increasing the humidification at the anode while decreasing the humidification at the cathode compared to the optimized condition when operating at a cell temperature of 70 °C (case of 70/70/65). Comparing the performance of the AEMFCs using 70/70/65 and 70/70/70, a slight improvement in the cell performance at a current density higher than 1150 mA cm^−2^ can be observed in the case using 70/70/65. This result could be attributed to the back-diffusion of water from the anode to the cathode which occurs in the cell using 70/70/65, partly relaxing the situation of the flooding at the anode and water scarcity at the cathode compared to the situation in the cell using 70/70/70. However, the back–diffusion rate of water through the membrane is still low compared to the water demand at the cathode. Omasta et al. [29] reported that the AEM with higher ionic conductivity can provide better water diffusivity through it due to its higher water uptake via the increase of ion-conducting groups (higher ion exchange capacity, IEC). Accordingly, the commercial membrane of AT-1 with an IEC of 1.0 mmol g^−1^, which is lower than other reported membranes, as summarized in Table 2, would have a lower diffusion rate of water across the membrane, resulting in an inadequate supply of water at the cathode.

## 4. Conclusions

The effects of cell temperature and the humidification of inlet reactant gases at different cell operating temperatures (60, 65, and 70 °C) on the AEMFCs were experimentally studied. Our results show that improvement in the cell performance at the higher cell operating temperature and appropriate humidification of the inlet gases can be largely achieved, owing to the higher reaction rate at the electrodes and enhanced ionic conductivity of the AEM, in addition to better water management. Furthermore, inlet gas humidification at both the anode and cathode inevitably affects the performance of the AEMFC at different cell temperatures because of the sensitivity of the AEMFC to the water distribution. The temperature gap between the optimized dew point and the cell temperature is enlarged at the anode (0, −3, and −5 °C) and reduced at the cathode (5, 2, and 0 °C) upon increasing the cell operating temperature (60, 65, and 70 °C, respectively). In addition, the effectiveness of back diffusion at supporting the water balance could depend on the properties of the AEM employed. Our findings of reaching a sophisticated water management can provide useful information for the development of high–performance AEMFC systems.

## Figures and Tables

**Table 1:** Physical properties of gas diffusion layer (GDL)-30.

Thickness (mm)	Polytetrafluoroethylene (PTFE) (wt.%)		Air Permeability (s)	Through Plane Resistance (mΩ cm^2^)	Mean Pore Diameter (µm)	Porosity (%)	Contact Angle (^o^)	
	Microporous Layer (MPL)	Back					MPL	Back
0.31	30	30	99.5	11.9	36.69	64.06	146.2	147.5

**Table 2:** Ion exchange capacity (IEC) and water uptake of some selected anion exchange membranes (AEMs).

Anion Exchange Membrane	IEC (mmol g^−1^)	Water Uptake (%)	Ref
AT-1	1.0	−	Manufacturer
Fumasep FAA-3-PK-75	1.39	20	Manufacturer
PPO-D20NC6NC6	1.94	61	[38]
RG-ETFE (30 kGy)-AEM	2.05	67	[29]
LDPE-g-VBC (50 µm)	2.3	70.8	[39]
